# Personality Variation in Little Brown Bats

**DOI:** 10.1371/journal.pone.0080230

**Published:** 2013-11-27

**Authors:** Allyson K. Menzies, Mary E. Timonin, Liam P. McGuire, Craig K. R. Willis

**Affiliations:** Department of Biology and Centre for Forest Interdisciplinary Research (C-FIR), University of Winnipeg, Manitoba, Canada; CNRS, Université de Bourgogne, France

## Abstract

Animal personality or temperament refers to individual differences in behaviour that are repeatable over time and across contexts. Personality has been linked to life-history traits, energetic traits and fitness, with implications for the evolution of behaviour. Personality has been quantified for a range of taxa (e.g., fish, songbirds, small mammals) but, so far, there has been little work on personality in bats, despite their diversity and potential as a model taxon for comparative studies. We used a novel environment test to quantify personality in little brown bats (*Myotis lucifugus*) and assess the short-term repeatability of a range of behaviours. We tested the hypothesis that development influences values of personality traits and predicted that trait values associated with activity would increase between newly volant, pre-weaning young-of-the-year (YOY) and more mature, self-sufficient YOY. We identified personality dimensions that were consistent with past studies of other taxa and found that these traits were repeatable over a 24-hour period. Consistent with our prediction, older YOY captured at a fall swarming site prior to hibernation had higher activity scores than younger YOY bats captured at a maternity colony, suggesting that personality traits vary as development progresses in YOY bats. Thus, we found evidence of short-term consistency of personality within individuals but with the potential for temporal flexibility of traits, depending on age.

## Introduction

Phenotypic variation within a species is a requirement for natural selection yet, in animal ecology and behaviour, intra-specific and intra-population variation have often been viewed as more of a nuisance than a biologically important phenomenon [1]. By definition, the study of animal personality, or consistent individual differences in behaviour, challenges this traditional view of variation, and is advancing our understanding of links between behaviour, physiology, and ecology [2]. Five ecologically relevant dimensions of personality have been well defined [2], including reaction to risky situations (i.e., shyness/boldness; e.g., [3], [4]), reaction to novel objects or situations (i.e., exploration/avoidance; e.g., [5]–[7]), activity levels (e.g., [8]), agonistic reactions to conspecifics (i.e., aggressiveness; e.g., [9], [10]), and non-aggressive reactions to the presence or absence of conspecifics (i.e., sociability; e.g., [11]). These inter-individual differences in behaviour tend to be repeatable across time, and can be heritable across generations [2], [6], [12], [13]. Recent studies have also shown that personality may impact life history and fitness via relationships to resource acquisition, reaction to predators, reproductive ability, and longevity [8], [13], [14], [15]. Variation in personality may also correlate with metabolism and energetics [16], although the ubiquity of these patterns and directions of relationships within species have not been fully established (e.g., [17]).

To be considered personality, individual differences in behaviour must be repeatable over time and/or across situations [2]. Repeatability represents the upper bound to heritability, and may be easier to measure than genetic relatedness in the field, so it can also be thought of as a first step towards determining if a behavioural phenotype is heritable [18], [19]. However, repeatability of many traits has not been well quantified for numerous species. For example, many studies have quantified repeatability of mating and courtship behaviours (e.g., mating calls/vocalization, mate choice) and anti-predator behaviour, but fewer have quantified repeatability of other personality traits, such as activity or exploration [12], especially in free-ranging mammals.

Although repeatability is fundamental to the definition of personality, ontogenetic or seasonal changes during development or reproductive cycles could cause within-individual variation in personality traits (e.g., activity, exploration propensity, boldness). This temporal variation would not necessarily preclude repeatability if between-individual variation at any point during the life cycle exceeded that within individuals or, in other words, the rank of individuals relative to one another remains consistent over time [20]. Especially pronounced shifts in personality traits have been observed at sexual maturity [20], [21], but development and reliance on parental care prior to sexual maturation could also influence within-individual variation. For species with precocial offspring, personality traits should remain relatively stable throughout ontogeny, as even young individuals of such species employ adult-like behaviours (e.g., [22], [23]). This consistency has been documented for a range of personality traits in precocial species (e.g., docility in ewes, [22]; boldness in deer, [23]; temperament and stress response in cattle, [24]). For altricial species, however, personality traits could change dramatically throughout development prior to sexual maturity as offspring acquire skills, exhibit more adult-like behaviours, and become self-sufficient (e.g., foraging behaviour in juvenile birds, [25]–[27]). A better understanding of within-individual variation in personality traits at different points in the life cycle, particularly for altricial mammals, is important because of the potential impact this variation can have on reproductive fitness and selection on behaviour [20].

A growing number of species are being studied in the context of personality, but there is still potential to diversify the field considerably. While the model organism approach has obvious value, the features which make a given species an ideal model for behavioural studies (e.g., readily captured or observed repeatedly, tractable in captivity), could also bias our understanding of personality variation [28]. One recent study examined “behavioural types” in big brown bats (*Eptesicus fuscus*; [29]) and bats, in general, have potential as another useful model for personality studies. Although many species will not be ideal for studies of long-term repeatability, as they are difficult to recapture in the wild, other aspects of their biology are highly amenable to comparative studies. For one, with approximately 1200 species, bats represent over 20% of extant mammals and exhibit large ecological and morphological diversity [30], which would allow for studies of personality variation across species in a phylogenetic and environmental context. Moreover, many species can be captured in large numbers, making them suitable for addressing questions requiring large sample sizes. As a result, they represent a potentially valuable taxon for studies of personality variation within species and for comparative studies addressing variation across species, similar to other better-studied taxa (e.g., non-human primates, [31]).

We quantified personality of the little brown bat (*Myotis lucifugus*). This species is one of the most common and widely distributed bat species in North America, although it is suffering high mortality in eastern North America due to the emerging disease white-nose syndrome [32]. Little brown bats hibernate for the winter in mines and caves in groups varying in size from a few to thousands of individuals [33]. In spring, females form maternity colonies where they give birth and care for highly dependent young, until these young-of-the-year (YOY) are capable of foraging on their own [34]. Males disperse individually or in small groups [33]. During the fall, prior to hibernation, large numbers of males, females, and independent YOY congregate in swarms outside hibernacula each night, presumably for mating and other social functions [34].

Our first objective was to test whether a modified version of the hole-board test, a standard open-field behavioural test commonly used to assess personality traits in rodents, would allow us to identify the same personality dimensions in bats as found in other taxa. We also aimed to quantify short-term repeatability of these traits by testing individuals on consecutive nights. We then used these data to test the hypothesis that personality traits may vary at sexual maturity and throughout the reproductive cycle by investigating whether the relative age of YOY or the reproductive status of adults influences variation in personality traits. Little brown bats are ideal for testing these hypotheses since they are an altricial mammal characterized by dramatic changes in the parental dependence of YOY between parturition and the first hibernation season. We predicted that older YOY bats captured during the fall swarming period would be more active and exploratory than younger YOY captured emerging from a maternity colony because YOY bats at mating swarms have reached independence and are preparing for hibernation.

## Materials and Methods

### Study Area and Subjects

Little brown bats were captured in Manitoba, Canada, during the summers of 2009 and 2010. In early August 2009, newly volant YOY bats (hereafter summer YOY) were caught emerging from a maternity colony in a building in Altamont, MB (49°21′N 98°35′W). In late August of 2009, older, presumably independent YOY (hereafter fall YOY) were captured during pre-hibernation swarming at Microwave Cave [35], a hibernaculum approximately 50 km north of Grand Rapids, MB (53°12′N 99°19′W). From June until August of 2010, reproductive and non-reproductive adult bats were captured at maternity colonies in both Altamont and Grand Rapids. The two field sites (Altamont and Grand Rapids) are approximately 400 km apart but bats at these two sites likely belong to the same population and experience similar conditions throughout the year. Based on our long-term banding studies of bats in Manitoba, individual little brown bats in this area routinely travel distances exceeding 500 km in one year [36], and there is very high gene flow among bats, and little evidence of population genetic structure at these sites (Martinez-Nunez and Willis, unpublished). Moreover, given the lack of suitable geology (i.e., limestone karst, abandoned mines) within hundreds of km of Altamont, individuals from the maternity roost there likely swarm at and hibernate in caves near Grand Rapids or other similar hibernacula in central Manitoba. Thus, there is a very high likelihood bats from both capture locations belong to the same population and experience similar conditions throughout their lives.

### Capture

All procedures and fieldwork were conducted under a Manitoba Conservation wildlife scientific permit (permit # WB11145), and were approved by the University of Winnipeg Animal Care Committee. We captured bats using harp traps and mist nets set at the entrance of the hibernaculum and maternity roost. Captured individuals were sexed, weighed to the nearest 0.1 g, and classified as adult or YOY based on the degree of ossification of the third metacarpal-phalangeal joint [37]. We also recorded forearm length and classified adult females as pregnant (presence of a foetus upon gentle abdominal palpitation), lactating (expression of milk and enlarged nipples surrounded by hairless patches) or non-reproductive. We used the residuals of a linear regression between mass and forearm length as a measure of body condition.

We transported bats to a field laboratory 10–40 km from the site of capture, where they were housed for up to 3 days. In all cases, the animals were housed in a dimly lit, quiet room, at room temperature, under natural photoperiod. We housed both summer and fall YOY bats in individual cloth bags (2009) and adult bats in wire-mesh cages (20 cm×25 cm×21 cm) covered with fabric, in groups of up to 6 individuals (2010). In both years, animals were handled minimally during the brief captivity period. We provided water via disposable pipette twice daily and fed bats mealworms (*Tenebrio molitor*) after their personality trials. In total, we captured 76 bats for behavioural testing (*n* = 28 YOY; *n* = 20 adult males; *n* = 28 adult females).

### Hole-board Test

**Figure 1:**
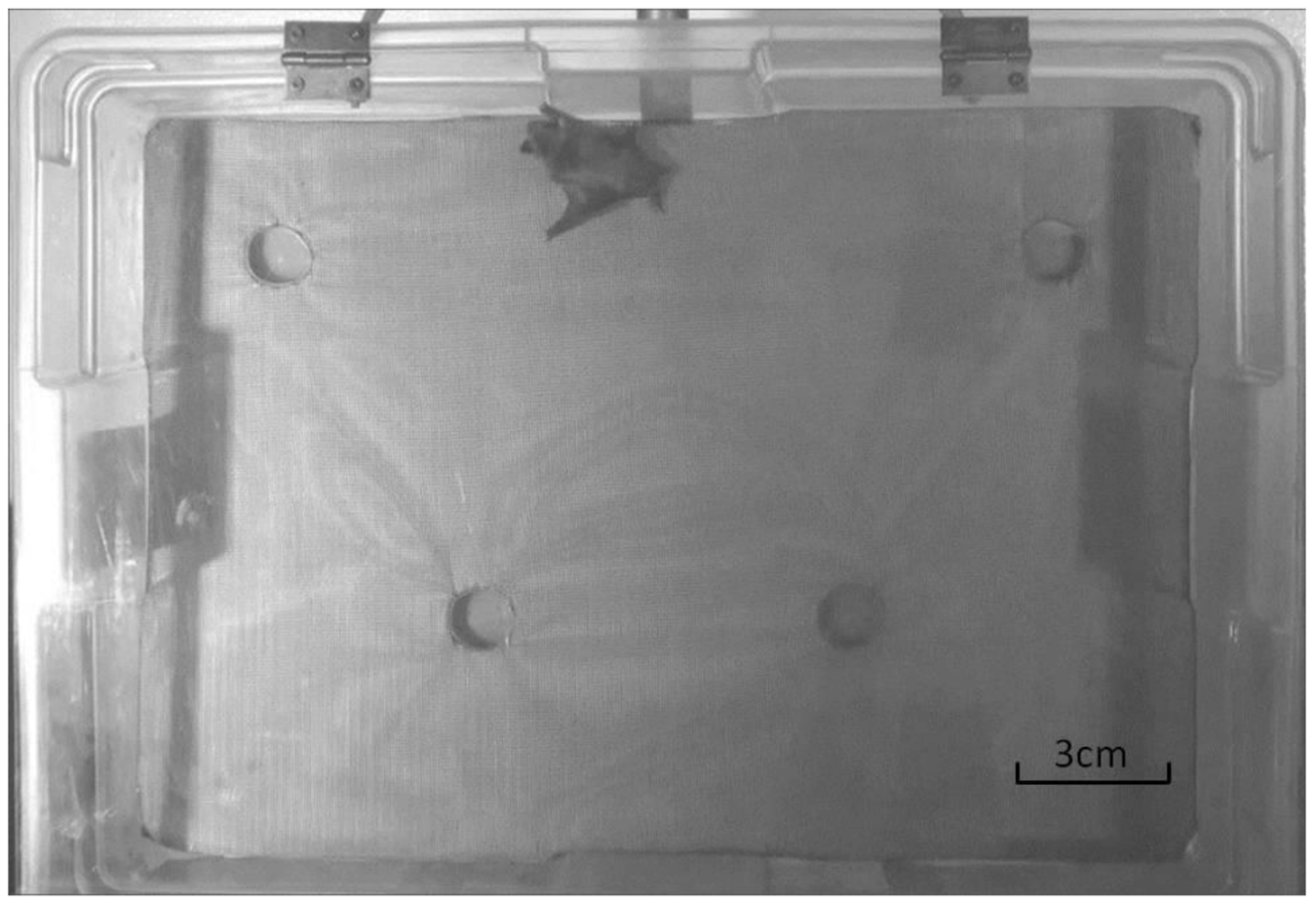
Screen capture of our modified hole-board test from a video recording of a behaviour trial.

Given that there are no standardized personality tests for bats, we chose to quantify activity and exploratory behaviour using a modified rodent hole-board test (e.g., [38]–[40]). Little brown bats are highly adept at climbing and crawling and must often crawl to explore cracks, crevices and other potential roost sites, so we reasoned that the hole-board test, with some minor modifications, would provide an ecologically relevant assessment of personality. The testing apparatus (Figure 1) was constructed to hang vertically, and consisted of a test chamber (57×42×14 cm) with a transparent cover and plastic window screening on the back surface to facilitate climbing by the bats. Four blind holes (2 cm deep by 3 cm diameter) were drilled into this surface. Two holes were positioned closer to the centre of the chamber (15 cm from nearest wall), while the other holes were positioned closer to the corners (5.5 cm from nearest wall). Investigation of the holes closer to the centre of the test is thought to indicate more thorough exploration and lower anxiety. A start box (14.5×14.5 cm) was attached to the bottom of the testing chamber, with a sliding door to separate the bat from the test until behavioural recording began.

Little brown bats are prone to enter torpor [41], so prior to behaviour testing we measured each individual’s body temperature by inserting a 1 mm diameter thermocouple probe 3 mm into the rectum. Bats were not tested until body temperature exceeded 30°C. Once normothermic, each bat was placed, individually, in the start box for two minutes before the sliding door was opened. Bats were given a maximum of one minute to enter the test on their own, after which they were gently pushed into the testing chamber with a smooth, plastic plunger. The sliding door was then closed to prevent re-entry. To simulate natural conditions, all trials were run in a darkened room at night, and the behaviour of each bat was recorded for 10 minutes using an infrared video camera (Sony AVCHD Handycam HDR-XR 550) mounted on a tripod. At the end of the trial, individuals were removed from the test and, to eliminate olfactory cues, the test was cleaned using mild, unscented dish detergent and water. All bats were fed mealworms and provided water after testing.

Videos were scored for a range of behaviours we selected based on previous studies of rodents (e.g., [39], [40]), their ecological relevance to bats, and our preliminary observations of bats in the hole-board test. We scored locomotion (proportion of time spent crawling or climbing in the test), frequency of flight attempts, the proportion of time spent echolocating (i.e., when the bats scanned the arena with their mouths open), frequency of head dips (the number of times an individual explored one of the holes either near the corner or the centre of the test, relevant to how bats might search potential roost openings in the wild), latency to head dip (the length of time it took each individual to first explore one of the holes), as well as latency to enter the test (the length of time it took each individual to enter the arena). We also measured the proportion of time spent grooming, as bursts of grooming are indicators of anxiety in rodents [42].

### Repeatability

Although it was not possible to capture the same individual bats over periods of weeks to months, we conducted two behavioural trials, separated by an interval of approximately 24-hours, to quantify short-term, within-individual repeatability. The second test for each individual was conducted under identical conditions, the night following the first behavioural test, controlling for factors likely to influence differences in behaviour among individuals (e.g., nutritional state assessed via body condition). At the end of the second set of behavioural trials, bats were released at their site of capture. To ensure that recaptured individuals could be identified, each bat was banded using lipped, numbered aluminum bands (Porzana Ltd., 2.9 mm, Icklesham, East Sussex, UK; 2009), or by subcutaneously injecting a passive integrated transponder (PIT) tag (Trovan Ltd. ID 100-01, Douglas, UK; 2010) prior to release.

### Statistical Analyses

All statistical analyses were conducted in R version 2.15.0 GUI 1.51 [43]. Principal Component Analysis (PCA) was used to reduce the number of measured behavioural traits into composite, synthetic variables or principal component (PC) scores (e.g., [17], [40]). An alternative method is Regularized Exploratory Factor Analysis (REFA), a data reduction method designed for small sample sizes. However, our dataset is well-suited to PCA (sample size larger than 50 individuals and relatively few variables included, [45]) so, for these reasons and to be consistent with previous studies addressing personality variation in wild-captured vertebrates (e.g., [8], [17], [40], [46]), we used PCA. All variables were scaled and centered (by subtracting the mean from each value and dividing this by the standard deviation) prior to inclusion in the PCA. We used the prcomp command in R, which generates PC scores based on singular value decomposition. In order to choose which principal components to retain, we used the Kaiser-Guttman criterion (eigenvalues >1; [44]) and a 10% minimum variance threshold for retention of a given component. To verify our choice of components, we also conducted a parallel analysis [47] using the paran package in R. PC scores from the PCA were used as representative personality scores in subsequent analyses.

We used intra-class correlation (ICC) to test for within-individual repeatability of PC scores and individual behaviours (e.g., [12]). The intra-class correlation coefficient is often used to assess reliability of measurements made by two different observers, or repeatability of traits within individuals, and is based on the mean squares as calculated for a standard ANOVA. It is calculated by dividing the variance among individuals (s^2^_a_) by the total variance (where s^2^ is the variance within individuals over time; [12], [18], [48]):
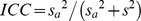



We used ANCOVA to determine effects of age, sex, body size, and body condition on PC scores. We tested the influence of these effects on PC scores from the first trial only, since trial one assessed the response to a novel environment. We did not include sampling location as a factor in our analyses since there is a very high likelihood that bats from both capture locations belong to the same population and experience similar conditions throughout their lives ( [36]; Martinez-Nunez and Willis, unpublished) and because relative age is the difference among the groups of YOY that we assumed would influence personality most strongly. We also omitted sampling year from the analysis because we captured exclusively YOY in 2009 and adults in 2010 so these variables were confounded. We also assumed that the effect of demographic (which has a large influence on most aspects of biology of bats) should be more pronounced. We used backwards, stepwise elimination to remove non-significant interactions and main effects until only significant terms remained (e.g. [8], [22], [46], [49]). To verify our findings we also compared a series of nested models. This analysis provided identical results as the step-wise model reduction so we only report results from the model reduction. We assessed the effect of relative age on PC scores of YOY bats and included sex, forearm length, and body condition as predictor variables. Forearm length and body condition differed between the sexes (female vespertilionid bats are often larger and in better body condition than males, [50]), thus we assessed the effect of reproductive status, body condition, and forearm length on PC scores of adult female bats, and the effect of forearm length and body condition on male bats, separately. After determining that there was no effect of either forearm length or body condition on either sex, we pooled females and males to determine the effect of sex on personality scores in all adult bats.

Levene’s tests were used to test for equality of variance among groups, Kolmogorov-Smirnov tests were used to test for normality, and data were log transformed as necessary to meet assumptions of parametric analyses. Significance for all tests was assessed at an alpha level of 0.05.

## Results

Three principal components each explained a minimum of 10% of the variance in recorded behaviours, met the Kaiser-Guttman criterion, and, cumulatively, explained over 60% of the total variance in the dataset (Table 1). The parallel analysis supported the first principal component (adjusted eigenvalue = 1.8) and provided weaker support for the second and third (adjusted eigenvalues = 0.8 in both cases). Locomotion, number of flight attempts, and echolocation contributed most strongly to the first component (PC1); individuals with high scores for PC1 had high levels of activity. Behaviours associated with exploration propensity contributed to PC2. Individuals with high scores for PC2 exhibited fast, superficial exploration, with shorter latency to enter the arena but longer latencies to head dip in the holes in the corners of the test. The third component (PC3) appeared to reflect anxiety and was represented most strongly by grooming as well as latency to head dip in the holes closer to the center of the arena.

**Table 1:** Results for Principal Component Analysis of behavioural responses of 76 little brown bats in a novel-environment test.

Variables	Components		
	1	2	3
Locomotion	**0.481**	−0.324	0.042
Echolocation	**0.422**	0.087	−0.096
Flight	**0.455**	0.171	−0.040
Latency to Enter	−0.339	**0.556**	−0.159
Latency to Head Dipin Holes (edge)	−0.237	−**0.714**	−0.092
Latency to Head Dipin Holes (centre)	−0.382	−0.192	**0.412**
Grooming	−0.257	0.052	−**0.886**
Standard Deviation	1.47	1.03	0.97
% of total variance	31.4	15.4	13.6
Cumulative Variance (%)	31.4	46.7	60.4

Principal Components retained met the Kaiser-Guttman criterion (i.e., eigenvalues >1, [44]). Bolded eigenvectors represent factors with loadings >0.4, which were considered to have contributed significantly to a particular component [40].

**Figure 2:**
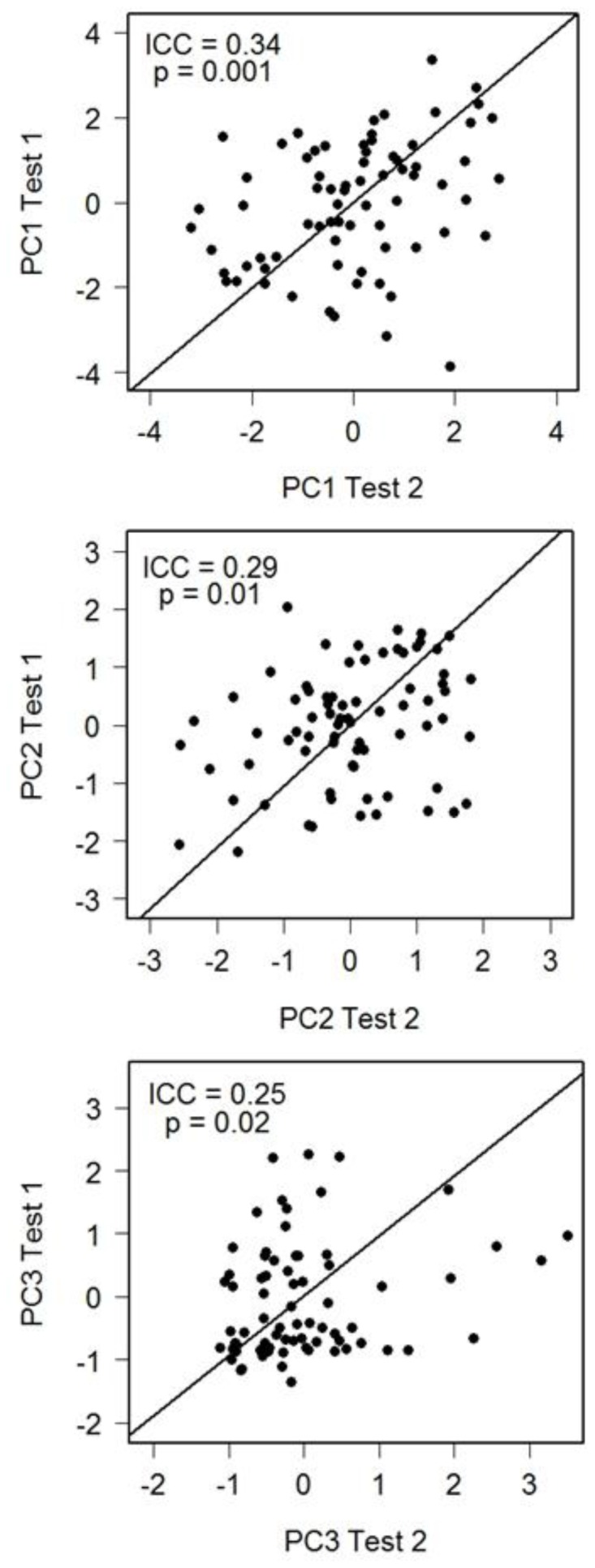
Scatterplots demonstrating repeatability of (a) PC1, (b) PC2 and (c) PC3 between behavioural trials 1 and 2 for 76 little brown bats in a novel environment test. Note that reduced-major-axis regression lines are plotted to illustrate the relationship, but repeatability was assessed using an intra-class correlation.

All PC scores were significantly repeatable from test 1 to test 2 (PC1, ICC = 0.34, d.f. = 73, *p* = 0.001; PC2, ICC = 0.29, d.f. = 73, *p* = 0.006; PC3, ICC = 0.25, d.f. = 73, *p* = 0.02; Figure 2). When we analyzed the individual behaviours that loaded strongly on PC1, PC2 and PC3, frequency of flights (ICC = 0.30, d.f. = 75, *p* = 0.004), time spent echolocating (ICC = 0.36, d.f. = 75, *p* = 0.001), latency to enter the test (ICC = 0.38, d.f. = 75, *p*<0.001), and latency to head dip in the holes closer to the corners of the test (ICC = 0.26, d.f. = 75, *p = *0.01) were significantly repeatable over the 24-hour interval.

**Figure 3:**
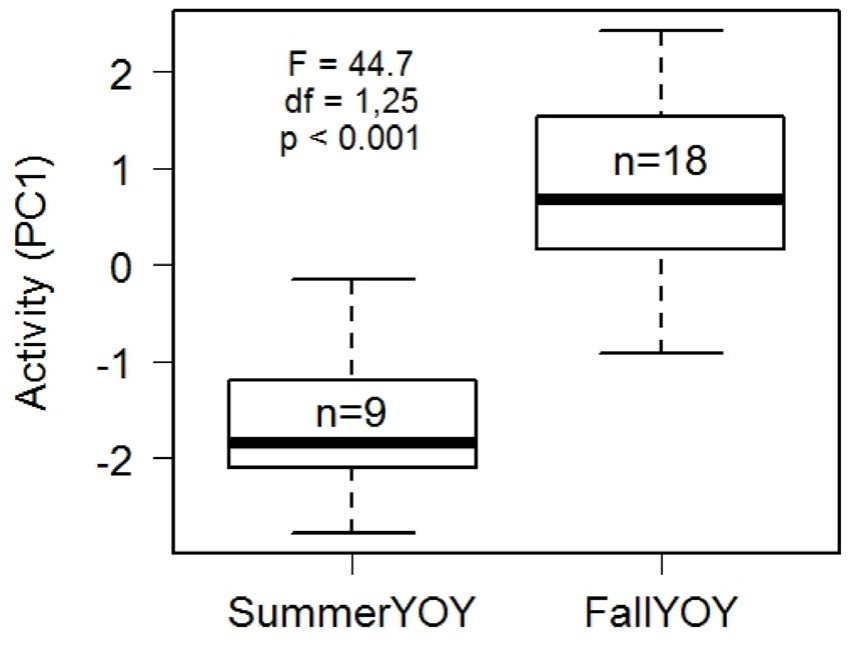
Comparison of activity levels for 28 YOY little brown bats captured at a maternity colony (summer YOY, n = 9) and a hibernaculum (fall YOY, n = 18) in August of 2009.

Fall YOY had significantly higher activity scores (PC1) than summer YOY (F_1,25_ = 44.7, p<0.001; Figure 3), with no effect of sex (F_1,23_ = 0.3, p = 0.59), forearm length (F_1,19_ = 0.3, p = 0.58), or body condition (F_1,18_ = 0.01, p = 0.98). Fall YOY exhibited significantly higher levels of all measures of activity during trial 1 (locomotion, *t* = 6.2, d.f. = 25, *p*<0.001; echolocation, *t* = 3.5, d.f. = 25, *p*<0.01; flights, *t* = 4.3, d.f. = 25, *p*<0.001). None of the factors in our models had a significant effect on PC2 (age, F_1,25_ = 2.1, p = 0.15; sex, F_1,17_ = 0.1, p = 0.72; forearm, F_1,19_ = 0.84, p = 0.37; body condition, F_1,21_ = 0.2, p = 0.70) or PC3 (age, F_1,25_ = 2.1, p = 0.16; sex, F_1,19_ = 0.1, p = 0.74; forearm, F_1,18_ = 0.8, p = 0.40; body condition, F_1,21_ = 0.2, p = 0.70) for YOY bats.

Reproductive status did not have an effect on any personality scores in adult females (PC1, F_2,22_ = 0.4, p = 0.67; PC2, F_2,22_ = 0.5, p = 0.59; PC3, F_2,22_ = 0.5, p = 0.62). There was also no effect of forearm length or body condition on PC1 (forearm, F_1,26_ = 4.0, p = 0.06; body condition, F_1,25_ = 3.6, p = 0.15), PC2 (forearm, F_1,25_ = 0.3, p = 0.61; body condition, F_1,26_ = 0.9, p = 0.35) or PC3 (forearm, F_1,26_ = 3.4, p = 0.08; body condition, F_1,25_ = 0.01, p = 0.92). Similarly, in adult males, there was no effect of body condition or forearm length on PC1 (forearm, F_1,18_ = 1.5, p = 0.24; body condition, F_1,17_ = 1.2, p = 0.29), PC2 (forearm, F_1,18_ = 0.8, p = 0.37; body condition, F_1,17_ = 0.9, p = 0.37) or PC3 (forearm, F_1,17_ = 2.0, p = 0.18; body condition, F_1,18_ = 0.9, p = 0.36). Finally, there was no effect of sex on PC1 (F_1,41_ = 2.5, p = 0.12), PC2 (F_1,41_ = 1.2, p = 0.29) or PC3 (F_1,43_ = 3.1, p = 0.09) of adult bats.

## Discussion

Based on our novel environment test, we found evidence for personality traits in little brown bats similar to those previously identified in rodents (e.g., [7],[40]), songbirds (e.g., [5], [6]) and fish (e.g., [51], [52]). In particular, behaviours reflecting activity, including locomotion, flight, and echolocation, separated into one component (PC1) while the second component (PC2) established a relationship between latency to enter the test and latency to head dip in holes closest to the walls of the test (i.e., holes 3 and 4). The hole-board test was initially developed to help separate activity components of personality in rodents from exploration components, which the standard novel environment test (i.e., without holes to investigate) failed to do [39]. We found that behaviours associated with exploration and activity separated into distinct components, suggesting that this test also isolates these personality dimensions in bats. The final two behaviours, grooming and latency to head dip in holes closer to the centre of the test (i.e., holes 1 and 2) grouped onto PC3. Although this link is unknown for bats, in rodents, bursts of grooming behaviour are a component of the stress response and indicate increased anxiety [42]. Latency to head dip in the holes that are more exposed (i.e., closer to the center of the arena) may also reflect anxiety as longer latencies for exploring these holes could indicate apprehension to venture away from the walls of the test. It would be useful to better define this personality dimension and assess whether these behaviours are associated with physiological correlates of stress (e.g., levels of circulating glucocorticoid hormones) in bats. These three components all satisfied the Kaiser-Guttman criterion, the objective criterion used in most other studies to retain or reject or reject personality dimensions (e.g., [8], [17], [40], [46]), as well as explaining a minimum of 10% of the total variation in our dataset. Strictly speaking, PC2 and PC3 fell just below the criterion for inclusion based on a parallel analysis (adjusted eigenvalue = 0.8), but given that they satisfied criteria used in other comparable studies and their clear biological significance we analyzed all three in more detail.

Individual bats reacted similarly during repeated tests and scores for all three principal components and were significantly repeatable over a 24-hour period (Figure 2). The intra-class correlation coefficients that we obtained (0.25–0.35) were comparable but slightly below the mean (r = 0.37) for published estimates for a wide range of behavioural traits in 114 species (reviewed by [12]). Our 24-hour inter-test interval fell within the range of published inter-test intervals for quantifying repeatability of behaviours in vertebrates (e.g., [53], [54]) but it was also relatively short. One potential limitation of novel environment tests repeated at short inter-test intervals is that some individuals may quickly habituate and change their behaviour between tests, while others might behave more consistently across trials. This individual variation in habituation behaviour reduces the potential to detect repeatability [55]. Based on our experience with bats, some individuals readily adapt to captivity and learn to eat novel food within 24 hours of capture, while others take significantly longer. As a result, some of the bats we tested may have habituated to the test quickly and responded differently in the second test causing us to underestimate trait repeatability [18]. In general, habituation is less likely over a longer inter-test time interval (e.g., a month or a year) compared to a shorter interval, reducing the within-individual variation between trials. Ideally, repeatability should be assessed over longer periods of weeks, months, or even years for long-lived species. Recapture rates are typically low for free-ranging bats and we were not able to recapture individuals in this study. For some bat species at some roosts it will be possible to recapture sufficient numbers of individual bats across longer intervals because individuals show high fidelity to specific sites throughout their lives (e.g., [36]). Holding bats in captivity for longer periods could also allow for longer intervals between tests although captivity could influence behaviour. We recommend both approaches for future studies.

We found evidence supporting the hypothesis that age of YOY influences variation in personality of wild-captured bats. Consistent with our predictions, fall YOY displayed elevated activity levels in conjunction with a critical shift in their independence and maturity (Figure 3). In a species that relies on flight, this shift could reflect morphological development (e.g., of the wings and pectoral muscles, which affect flight ability, [26]). However, we found no difference in body mass, forearm length or body condition between the two groups which indicates that volant YOY from both groups had reached adult size when we assessed their behaviour. Thus, higher activity levels of fall YOY could reflect an ontogenetic shift in behaviour as these bats became independent. Summer YOY were captured at the natal roost, and were likely still reliant on their mothers and not yet self-sufficient. In contrast, fall YOY were presumably independent, as they had already left their maternity colony and traveled to a hibernaculum (which can be 30–500 km from the natal roost in our study area; [36]). Fall YOY would also need to have more developed echolocation, one of the measures of activity that contributed to PC1, because bats rely on echolocation for foraging and navigation (e.g., [56], [57]). These results support our prediction that YOY would show increased activity in conjunction with this critical shift in development.

Another possibility is that the difference between summer and fall YOY reflects links between life history, ecology and personality. The “fast”, highly active individuals may have also been quick to reach independence and disperse to swarming sites while the “slower”, less active individuals may have taken longer to become independent, and remained at the maternity roosts for longer. Previous studies have demonstrated that activity in novel environments is correlated with dispersal in the field (e.g., *Parus major*, [58]) and that dispersal latency decreases with increasing exploratory activity (e.g., *Mus musculus musculus*, [59]), which is consistent with our results. More active YOY may have dispersed from the natal roost and ventured to swarming sites earlier. Taken together, these findings highlight an important consideration for studies of personality that address behavioural differences of different age classes of animals. Grouping all YOY bats together, regardless of developmental stage, would have masked subtle but potentially important differences in behaviour within this age class.

We found evidence that personality traits in bats differentiate into similar components as those described in past studies of rodents, fish, and birds, and that the open-field component of the hole-board test was useful for measuring activity and exploration. Other standardized behavioural tests are needed to validate results found in this study and to further examine a range of personality traits in this taxon. We also found evidence of temporal flexibility in activity of YOY bats. Although most studies control for the age of subjects, typically all YOY are considered as one discrete group. Our results indicate that important biological differences exist within this category. Since within-individual variation of personality traits in bats has received relatively little attention, further studies are necessary to understand both the consistency and temporal flexibility of personality in bats. Future studies investigating consistency, or inconsistency, of personality traits throughout energetically demanding portions of the life cycles (i.e., mating/reproduction, preparing for hibernation) for both adult male and adult female bats may also help shed light on potential links between personality and energetics.
